# Characterization of ABC Transporters in EpiAirway™, a Cellular Model of Normal Human Bronchial Epithelium

**DOI:** 10.3390/ijms21093190

**Published:** 2020-04-30

**Authors:** Bianca Maria Rotoli, Amelia Barilli, Rossana Visigalli, Francesca Ferrari, Caterina Frati, Costanza Annamaria Lagrasta, Maria Di Lascia, Benedetta Riccardi, Paola Puccini, Valeria Dall’Asta

**Affiliations:** 1 Laboratory of General Pathology, Department of Medicine and Surgery, University of Parma, Via Volturno, 39, 43125 Parma, Italy; amelia.barilli@unipr.it (A.B.); rossana.visigalli@unipr.it (R.V.); francesca.ferrari@unipr.it (F.F.); valeria.dallasta@unipr.it (V.D.); 2 Pathology Unit, Department of Medicine and Surgery, University of Parma, Via Gramsci, 14, 43126 Parma, Italy; caterina.frati@unipr.it (C.F.); costanzaannamaria.lagrasta@unipr.it (C.A.L.); 3 Preclinical Pharmacokinetics, Biochemistry & Metabolism Dept., Chiesi Farmaceutici, Largo Francesco Belloli, 43122 Parma, Italy; M.dilascia@chiesi.com (M.D.L.); B.Riccardi@chiesi.com (B.R.); P.Puccini@chiesi.com (P.P.)

**Keywords:** respiratory pharmacology, drug delivery, translational pharmacology, ATP-binding cassette transporters

## Abstract

The ATP-binding cassette (ABC) transporters P-glycoprotein (MDR1/*ABCB1*), multidrug resistance-associated protein 1 (MRP1/*ABCC1*), and breast cancer resistance protein (BCRP/*ABCG2*) play a crucial role in the translocation of a broad range of drugs; data about their expression and activity in lung tissue are controversial. Here, we address their expression, localization and function in EpiAirway™, a three-dimensional (3D)-model of human airways; Calu-3 cells, a representative in vitro model of bronchial epithelium, are used for comparison. Transporter expression has been evaluated with RT-qPCR and Western blot, the localization with immunocytochemistry, and the activity by measuring the apical-to-basolateral and basolateral-to-apical fluxes of specific substrates in the presence of inhibitors. EpiAirway™ and Calu-3 cells express high levels of MRP1 on the basolateral membrane, while they profoundly differ in terms of BCRP and MDR1: BCRP is detected in EpiAirway™, but not in Calu-3 cells, while MDR1 is expressed and functional only in fully-differentiated Calu-3; in EpiAirway™, MDR1 expression and activity are undetectable, consistently with the absence of the protein in specimens from human healthy bronchi. In summary, EpiAirway™ appears to be a promising tool to study the mechanisms of drug delivery in the bronchial epithelium and to clarify the role of ABC transporters in the modulation of the bioavailability of administered drugs.

## 1. Introduction

ATP-binding cassette (ABC) transporters are a superfamily of transmembrane proteins that can transport a wide variety of substrates, including endogenous molecules and xenobiotics, in an energy-dependent manner [1]. In particular, the three ABC transporters P-glycoprotein (MDR1, *ABCB1*/MDR1), multidrug resistance-associated protein 1 (*ABCC1*/MRP1), and breast cancer resistance protein (*ABCG2*/BCRP) play a crucial role in the translocation of a broad range of drugs, such as several chemotherapeutic agents [2]; their overexpression is one of the mechanisms responsible for the development of multiple drug resistance by cancer cells [3].

Literature evidence indicates that they are expressed in lung tissue at different levels [2,4,5], suggesting a potential role for these proteins in the delivery of respiratory drugs, including corticosteroids, β_2_-adrenergic agonists, mucolytic and anticholinergic agents [5]. This evidence, along with the recent developments in delivering drugs to the lung, emphasize the need for robust and reproducible in vitro cell models to predict the fate of inhaled medicines in vivo.

Respiratory epithelial cell lines of human origin such as Calu-3, 16HBE14o-, NCl-H441 and BEAS-2B are usually employed as reference models in studies of pulmonary drug absorption, but criticisms have been raised about their reliability as models for normal airways. Indeed, although these cells are highly versatile and, thus, widely employed for studies of drug absorption, they all correspond to immortalized or transformed cell lines with altered genetic profiles that likely differ from that of normal epithelial cells [6,7]. To bypass this issue, in vitro models that more closely mimic the biology of normal airway epithelium in vivo have been employed. Among them, normal human bronchial epithelial cells (NHBE) appeared promising, since they can form a pseudostratified cell layer, including ciliated and goblet cells, that nearly resembles normal airways [8,9,10]; however, at present, there is no universally accepted protocol for the culture of these cells and transport data in NHBE layers appear poorly reproducible [8,10].

More recently, three-dimensional (3D)-constructs of epithelial tissues have been developed that accurately reproduce the physiology of normal human airway epithelia. These ready-to-use models, known by the brand names EpiAirway™ (MatTek Corporation) and MucilAir™ (Epithelix), are composed of primary human epithelial cells freshly isolated from bronchial biopsies and include a functional mucociliary system. Although most of the studies thus far performed on EpiAirway™ mainly focused on their biological responses to air pollution [11], the same system also proved a good tool for estimating the permeability of different passively transported drugs [12,13]. To date, however, a precise description of ABC transporters specifically expressed and active in this cell model is still lacking.

Hence, the aim of our study has been to perform a characterization of the three transporters MDR1, MRP1 and BCRP in terms of expression, localization and activity in EpiAirway™ cells; Calu-3 cells, one of the most representative cell model of human bronchial epithelium, has been used for comparison.

## 2. Results

**Figure 1:**
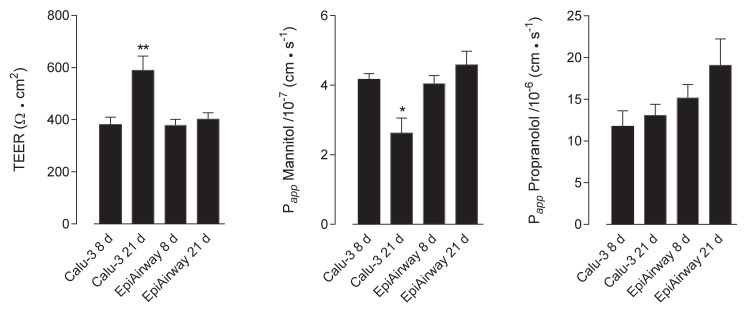
Trans-epitelial electrical resistance (TEER) and cellular permeability (*P_app_*) in EpiAirway™ and Calu-3 cells. TEER values and *P_app_* of mannitol and propranolol were measured in cells cultured under air–liquid interface (ALI) conditions for 8 d or 21 d, as indicated (see Methods). Bars represent the mean ± standard error of the mean (SEM) of five (TEER), four (mannitol) and three (propranolol) independent determinations. * *p* < 0.05, ** *p* < 0.01 vs. other models.

In order to verify the barrier properties of Calu-3 and EpiAirway™, the integrity of the monolayers was preliminary addressed, both in “early” differentiated cultures (maintained under air–liquid interface (ALI) conditions for 8 d) and “fully” differentiated (maintained at ALI for 21 d). To this end, the trans-epithelial electrical resistance (TEER) of the monolayers has been measured, while their para- and trans-cellular permeability has been evaluated by analyzing the fluxes of mannitol [14,15] and propranolol [16], respectively. As shown in Figure 1, no difference was observed in terms of TEER and mannitol permeability values among EpiAirway™ at the different lengths of culture; a significant increase occurred, instead, in Calu-3 cells after 21 d at ALI, where, consistently, the P*_app_* for mannitol decreased. As for propranolol, the permeability of EpiAirway™ appeared slightly higher than that of Calu-3 cells at both times of culture, suggesting that the normal tissue is likely more prone to transcellular fluxes due to passive diffusion.

**Figure 2:**
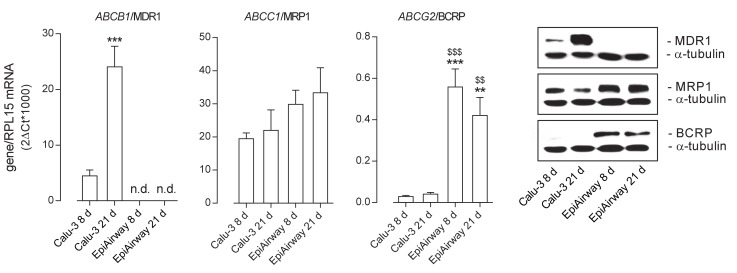
Expression of ATP-binding cassette (ABC) transporters in Calu-3 cells and EpiArway™ cultured under ALI conditions for 8 d or 21 d. Left: the mRNAs for the genes of interest were determined by means of RT-qPCR analysis and normalized for that of the reference gene (*RPL15*), according to the formula described in Methods. Data are means ± SEM of three independent experiments. ** *p* < 0.01, *** *p* < 0.001 vs. Calu-3 8 d; ^$$^ *p* < 0.01, ^$$$^ *p* < 0.001 vs. Calu-3 21 d; n.d. not detectable. Right: the expression of the transporters at the protein level was addressed by means of Western blot analysis, as described in Methods. A representative blot is shown, repeated three times with comparable results. MDR1: P-glycoprotein; MRP1: multidrug resistance-associated protein 1; BCRP: breast cancer resistance protein.

The expression of the ABC transporters *ABCB1*/MDR1, *ABCC1*/MRP1, and *ABCG2*/BCRP was then evaluated at both the mRNA and protein level in the two cell models (Figure 2). As for MDR1, its amount markedly increased in Calu-3 cells along with the culture time, with the gene expression at least quadruplicating after 21 d at ALI and the protein band, faint at 8 d, strongly evident at 21 d; on the contrary, no expression of the transporter was observed in EpiAirway™ at any time of culture, neither as mRNA nor as protein. *ABCC1*/MRP1 was readily detectable in both cell models, although slightly more abundant in EpiAirway™. Lastly, the mRNA for *ABCG2* was higher expressed in EpiAirway™ than in Calu-3 cells, with no significant difference between the culture times; consistently, the band corresponding to BCRP was absent in Calu-3 cells while readily detectable in EpiAirway™.

**Figure 3:**
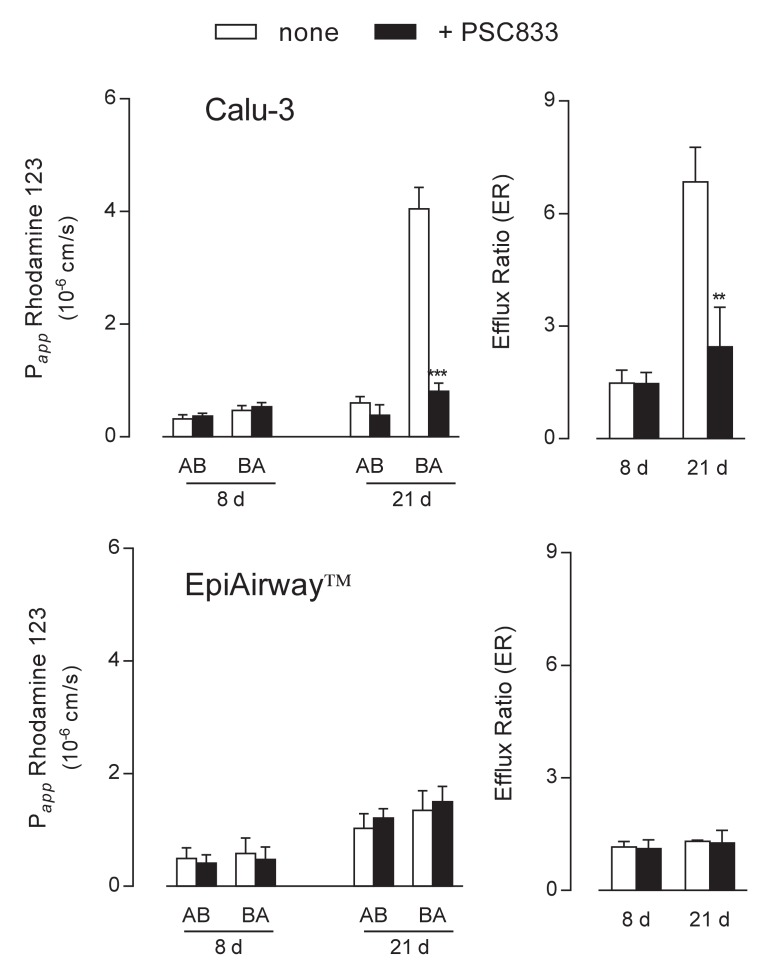
MDR1 activity in Calu-3 cells and EpiArway™ maintained under ALI conditions for 8 d or 21 d. The apical-to-basolateral (AB) and basolateral-to-apical (BA) fluxes of 1 µM Rhodamine 123 were monitored both in the absence (none) and in the presence of 10 µM PSC833, as indicated. Data obtained were employed to calculate the efflux ratio (ER), as defined in Methods. Bars represent the mean ± SEM of three independent experiments. ** *p* < 0.01, *** *p* < 0.001 vs. none.

The functional activity of the transporters was next addressed by monitoring the apical-to-basolateral (AB) and basolateral-to-apical (BA) fluxes of tracers specific for each protein. To this end, fluorescent Rhodamine 123 was employed as substrate of MDR1 [17,18] to evaluate the activity of the transporter in the two cell systems. Results presented in Figure 3 were consistent with data obtained from the analysis of gene and protein expression. In Calu-3 cells grown at ALI for 8 d, the P*_app_* of Rhodamine 123 for AB and BA fluxes were comparable and insensitive to the presence of PSC833, described as an inhibitor of MDR1 [19,20], indicating a negligible activity of the transporter under this condition. Conversely, after 21 d at ALI, the P*_app_* for BA transport was hugely higher than that of AB flux and almost completely hindered by PSC833, resulting in an efflux ratio (ER) value that decreased from 7 in control cells to 2.8 in the presence of the inhibitor. As a result, we can confirm that MDR1 in Calu-3 cultures is expressed and operative on the apical membrane of the cells only after 21 d at ALI. When addressing the activity of the transporter in EpiAirway™, no differences were observed between the AB and BA fluxes of Rhodamine 123 at both culture times; this result, along with the lack of inhibition by PSC833, further sustains the absence of MDR1 in this cell system in line with data of mRNA and protein expression.

**Figure 4:**
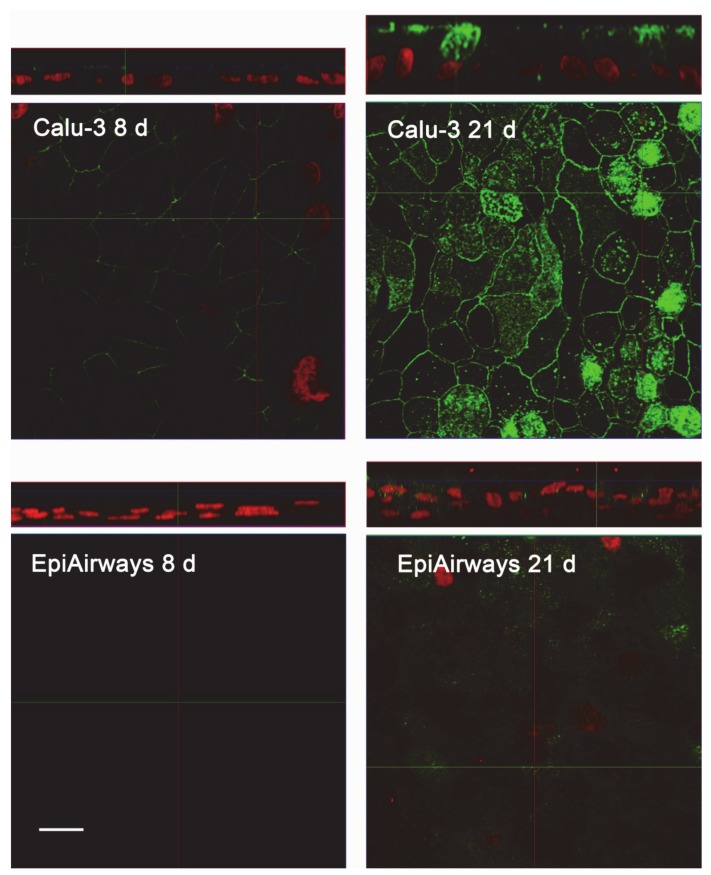
Immunocytochemical analysis of MDR1 expression in Calu-3 cells and EpiArway™. The expression of the transporter was evaluated in cultures maintained under ALI conditions for 8 d or 21 d by means of confocal laser scanning microscopy. Green signal: MDR1 immunolabeling; red signal: nuclear staining with propidium iodide. A single XY scan is shown, along with the XZ section of the plane (top). Scale bar = 10 µm.

**Figure 5:**
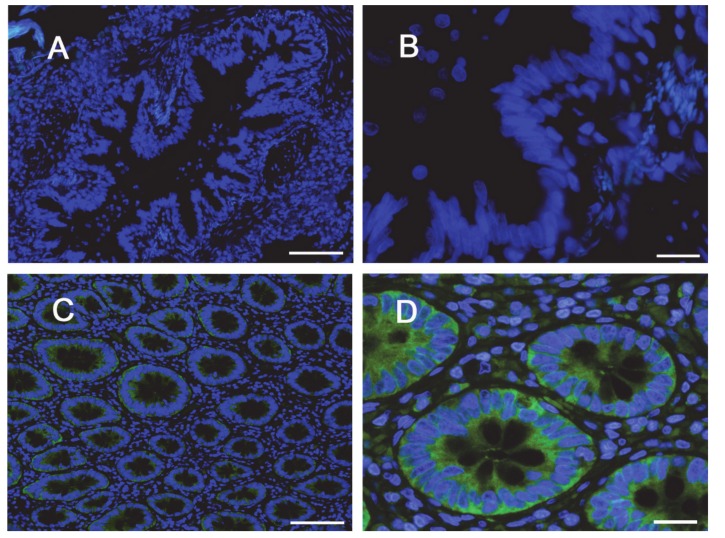
Immunohistochemical (IHC) detection of MDR1 in human bronchial and intestinal epithelium. Paraffin sections of bronchus (panels **A**,**B**) and colon (panels **C**,**D**) specimens from healthy subjects were immunostained with anti-MDR1 antibody (green) and counterstained with 4′,6-diamidino-2-phenylindole, DAPI (blue). Scale bars: (**A**–**C**) = 100 µm; (**B**–**D**) = 20 µm.

The same conclusions were reached when addressing protein expression by means of confocal immunocytochemistry. Images in Figure 4 show a faint staining of MDR1 in Calu-3 cultured at ALI for 8 d that became readily appreciable on the apical membrane of the monolayer when the cultures were grown for longer time. No staining of the transporter was detectable in EpiAirway™ at any culture time, excluding once again the presence of MDR1 in these cells. To verify whether this finding really reflects the pattern of expression of the transporter in human airways in vivo, we next checked the expression of MDR1 in paraffin-embedded specimens of normal human bronchi (Figure 5A,B) and colon epithelium (Figure 5C,D), employed as positive control [21,22]; the results obtained definitely confirmed the absence of the protein in respiratory epithelium.

**Figure 6:**
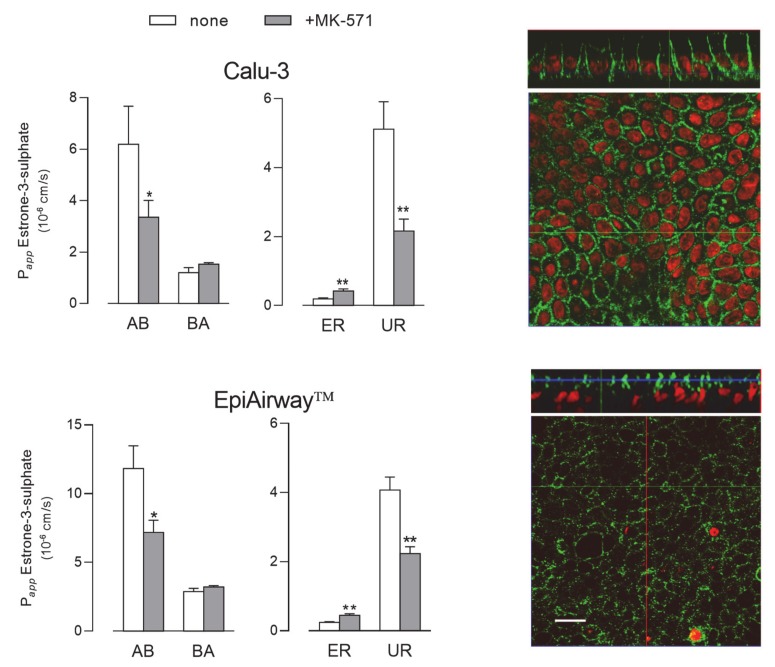
MRP1 activity and expression in Calu-3 cells and EpiArway™ cultured under ALI conditions for 21 d. Left: the apical-to-basolateral (AB) and basolateral-to-apical (BA) fluxes of ^3^H-estrone-3-sulphate (0.5 µM, 3 µCi/mL) were monitored both in the absence (none) and in the presence of 50µM MK-571, as indicated. Data obtained were employed to calculate the efflux (ER) and uptake (UR) ratio, as defined in Methods. Bars represent the mean ± SEM of three independent experiments. * *p* < 0.05, ** *p* < 0.01 vs. none. Right: the expression of the transporter was evaluated by means of confocal laser scanning microscopy. Green signal: MRP1 immunolabeling; red signal: nuclear staining with propidium iodide. A single XY scan is shown, along with the XZ section of the plane (top). Scale bar = 10 µm.

Next, we evaluated the activity of MRP1 by monitoring the fluxes of ^3^H-estrone-3-sulphate [23], either in the absence or in the presence of MK-571, described as a good inhibitor of the transporter [18,24] (Figure 6). Since no differences in terms of mRNA or protein level occurred between cells maintained for 8 d and 21 d, the functional analysis was performed only in Calu-3 cells and EpiAirway™ grown at ALI for 21 d. In both cell systems the P*_app_* for AB flux was markedly higher than that for BA, yielding values of uptake ratio (UR) much higher than ER and, hence, suggesting that the transporter mainly operates at the basolateral side of the monolayers; the inhibitory effect of MK-571 on AB, but not on BA flux, further confirmed this finding. In line with these results, the images obtained with confocal microscopy also showed a clear-cut distribution of the protein staining at the basolateral side of both Calu-3 and EpiAirway™ cultures.

**Figure 7:**
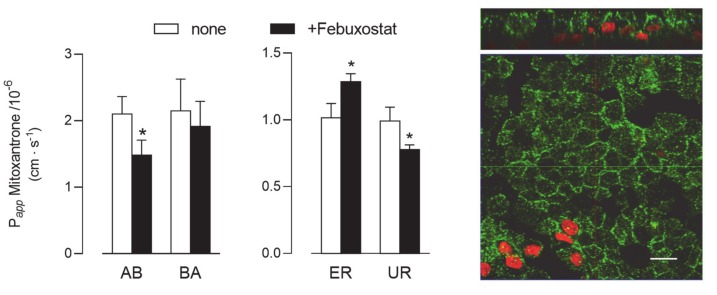
BCRP activity and expression in EpiArway™ cultured under ALI conditions for 21 d. Left: the apical-to-basolateral (AB) and basolateral-to-apical (BA) fluxes of ^3^H-mitoxantrone (0.1 µM, 1 µCi/mL) were monitored both in the absence (none) and in the presence of 10 µM Febuxostat, as indicated. Data obtained were employed to calculate the efflux (ER) and uptake (UR) ratio, as defined in Methods. Bars represent the mean ± SEM of three independent experiments. * *p* < 0.05 vs. none. Right: the expression of the transporter was evaluated by means of confocal laser scanning microscopy. Green signal: BCRP immunolabeling; red signal: nuclear staining with propidium iodide. A single XY scan is shown, along with the XZ section of the plane (top). Scale bar = 10 µm.

Lastly, given the absence of BCRP protein in Calu-3 cells (see Figure 2), the activity of this latter transporter was addressed only in EpiAirway™ cultured at ALI for 21 d. As shown in Figure 7, P*_app_* values for AB and BA fluxes of ^3^H-mitoxantrone, employed as a tracer [25], were identical, reflecting comparable values for ER and UR and thu excluding a definite directionality of the transport. However, a modest but significant decrease of P*_app_* for AB flux was observed upon the addition of a BCRP specific inhibitor, Febuxostat [26,27], while BA flux remained almost unaffected; the resulting slight decrease of UR is consistent with activity of the transporter at the basolateral side of the monolayer. BCRP staining by means of immunocytochemistry revealed the presence of the protein on both the apical and lateral layers.

## 3. Discussion

In vitro cell models are fundamental tools for the development and screening of drugs in pre-clinical studies. Although the only US Food and Drug Administration (FDA)-approved predictive model for studies of drug absorption is the intestinal Caco-2 cell line [28], airway epithelial Calu-3 cells represent one of the most employed systems for the prediction of drug absorption in the lung [29,30,31,32]. The convenience, low cost and robustness of this cell line make it a good model for the screening of drug permeability in the airways [29], and the expression and activity of ABC transporters in these cells have been widely investigated in recent years (for a review, see [4]). However, the need for more bio-relevant models exhibiting normal human tissue structure and cellular morphology remains pressing.

Recently, the EpiAirway™ system, along with MucilAir™ bronchial tissues (Epithelix), have gained particular attention for being very close to the normal human bronchial epithelium in terms of cell type composition and histology. In a recent study, EpiAirway™ was used to estimate the permeability of model compounds across cells so as to clarify their fullness as an estimation system for nasal drug absorption in rats [13]; nevertheless, pharmacological data in this model are still incomplete and the expression and activity of the different transporters, as well as their contribution to drug absorption is thus far uncharacterized. To address this issue, we carried out a detailed characterization of ABC transporters *ABCB1/*MDR1, *ABCC1*/MRP1 and *ABCG2/*BCRP in EpiAirway™ by addressing their expression, localization and activity; Calu-3 cells have been employed for comparison. The results we obtained clearly demonstrate the presence of both MRP1 and BCRP in EpiAirway™, while exclude the expression and activity of MDR1 in this cell system.

As for MRP1, the expression of the protein was readily detectable in EpiAirway™ at levels comparable to those of Calu-3 cells. This finding is consistent with literature studies showing a clear-cut expression of this transporter in both Calu-3 and BEAS-2B cells [18,33]; similarly, Berg et al. described high expression of *ABCC1/*MRP1 mRNA in bronchial specimens from human lung explants, both in central airways and peripheral region [34]. Our immunocytochemical analysis clearly visualized the transporter on the basolateral membrane of both EpiAirway™ and Calu-3 cells; consistently, bidirectional transport studies proved the efficacy of the MK-571 inhibitor on the sole apical-to-basolateral flux. The same localization has been previously reported for MRP1 in Calu-3 cells [18] and in several respiratory cell culture models in vitro, as well as in lung tissues [4], pointing to a physiological role for this transporter in reducing drug absorption by extruding potentially harmful substances out of the epithelium/cells towards the bloodstream.

The BCRP transporter is known to be involved in cell resistance to toxins and several chemotherapeutic agents (e.g., mitoxantrone). Data regarding its expression in the lung are sparse and not univocal: the mRNA for the transporter has been described as barely detectable in healthy lung tissues [34]; as far as the protein is concerned, cytoplasmic staining of BCRP has been observed by Fetsch et al. only in alveolar pneumocytes [35], while low but detectable amounts of the transporter have been reported in the epithelial layer and in the seromucinous glands of normal lung tissues [36]. More recently, a quantitative analysis of the protein expression profile has confirmed the presence of BCRP in human lung tissues, as well as in normal human bronchial epithelial cells (NHBEs) [37]. According to our results, no expression of the protein was observed in Calu-3 cells, despite a modest amount of mRNA being present. Although BCRP has been detected in non-polarized cultures of Calu-3 grown on plasticware [38], findings are consistent with literature data indicating that the expression of the transporter is low in Calu-3 and high in 16HBE14o- cells [39]. As for EpiAirway™, our data indicate that this model expresses BCRP at high levels with a heterogeneous staining of both the lateral and apical membranes. Consistently, the P*_app_* values for mitoxantrone, comparable in the two AB and BA directions, suggest a widespread distribution of the protein on cell borders; however, since the addition of the inhibitor hinders only the apical-to-basolateral flux, the activity of the transporter is supposed to be predominantly localized on the lateral side of cell monolayers. Further investigation will be required to clarify this issue.

Lastly, MDR1 is a glycoprotein widely expressed on the apical membrane of different tissues. As for in vitro models of pulmonary epithelium, a clear dependence of the expression and function of MDR1 on the culture time and passage number has been established for polarized monolayers of Calu-3 cells [8,10,17]. Consistently, we observed only barely detectable levels of the transporter in Calu-3 cells after 8 d at ALI, while a much higher amount of the protein was evident at the apical side of “late” cultures. Literature data about MDR1 expression in the respiratory epithelium indicate expression levels ranging from low to moderate (although still lower than in other tissues, such as the kidney, liver, or intestine [5]). Data obtained by Madlova et al. with digoxine fluxes in normal human bronchial cells (NHBE) were consistent with low levels of MDR1 activity at the basolateral side of the cells after 14-21 d culture [10]; the transporter was undetectable in tracheal–bronchial BEAS2B, bronchiolar–alveolar NCI-H292 and NCI-H441 cells, as well in primary cultured human lung cells from trachea and bronchi [33]. The issue is more controversial as far as normal human lung tissues are concerned. Berg et al. reported a low expression of *ABCB1/*MDR1 mRNA in the central and peripheral region of the lung from both healthy and chronic obstructive pulmonary disease (COPD) subjects [34]; the same author, however, more recently showed MDR1 protein expression in pulmonary lung epithelium, with more profound staining on the apical cell membrane of ciliated cells as well as in the bronchiolar epithelium [40]. Consistently, Scheffer et al. showed the staining of MDR1 on the apical membrane of bronchial and bronchiolar epithelium and, to some degree, in alveolar macrophages [36]. In our hands, no expression of the transporter was detected in EpiAirway™, either at the mRNA or protein level, not even when the cell culture was prolonged to 21 d. Consistently, the measurement of Rhodamine 123 fluxes in these cells resulted in very low values of both P*_app_* and ER that were not affected by the addition of the inhibitor PSC833, hence excluding the contribution of active transporters. In further support of this finding, our immunohistochemistry results clearly evidenced the absence of staining of MDR1 protein in specimens of bronchial epithelium from healthy subjects. In light of the relevance of the issue, we are aware that the differences between our findings and the literature deserve to be better investigated. To this concern, the possibility exists that the discrepancies with literature data can be referred to the different antibodies employed; in our hands, the reliability of the antibody is sustained by the strong positivity of MDR1 staining in colon epithelium, employed as a positive control [21,22].

In summary, our characterization of ABC transporters in EpiAirway™ demonstrates the presence of both MRP1 and BCRP in this cell system, and the lack of MDR1 (both expression and activity). Since this latter transporter, readily detectable in Calu-3 monolayers, is lacking in specimens from human healthy bronchi, we suggest that EpiAirway™ better reflects the phenotype of normal human bronchial epithelium in vivo. Our findings hence sustain the reliability of this 3D-cell system as a promising tool to study the mechanisms of drug delivery in human airways.

## 4. Material and Methods

### 4.1. Cell Cultures

The EpiAirway™ system (AIR-200-PE6.5) was provided by MatTek Corporation (Ashland, MA, USA) as “early” differentiated (8 d after seeding) or “fully” differentiated (21 d after seeding) cells. Cultured on polyethylene terephthalate (PET) membrane inserts at the air–liquid interface (ALI), EpiAirway™ recapitulates aspects of the in vivo microenvironment of the lung; this system is produced from primary human tracheal–bronchial epithelial cells that form a fully differentiated, pseudostratified columnar epithelium containing mucus-producing goblet cells, ciliated cells and basal cells. Upon arrival, tissue inserts were transferred to 24-well plates containing 600 µL of the AIR 200-M125 medium and equilibrated overnight at 37 °C and 5% CO_2_. Cultures from five different healthy donors were employed.

Calu-3 cells from a human lung adenocarcinoma (American Type Culture Collection) were grown in Eagle’s Minimum Essential Medium (EMEM), as previously described [41], and employed between 25–32 passages. For culture under ALI conditions, 1 × 10 ^5^ cells were seeded onto Transwell PET inserts (0.33 cm^2^, 0.4 µm pore size; Falcon) and allowed to differentiate for 8 d or 21 d, as required. The apical medium was removed 24 h after seeding, while the basolateral medium was renewed every other day.

### 4.2. Trans-epithelial Electrical Resistance (TEER) and Cellular Permeability

The integrity of the cell monolayer was verified by measuring the trans-epithelial electrical resistance (TEER) with an epithelial voltmeter (EVOM, World Precision Instruments).

In parallel, the paracellular and transcellular permeability of cultures to solutes was assessed by monitoring the apical-to-basolateral fluxes of ^14^C-mannitol and ^3^H-propranolol, respectively. In detail, cell monolayers were washed and incubated for 30 min in Hank’s Balanced Salt Solution (HBSS, pH 7.4, 37 °C); HBSS containing ^14^C-mannitol (1 µCi/mL, corresponding to 20 µM) or ^3^H-propranolol (3 µCi/mL; 10 µM) was then added to the apical side (donor chamber) of the monolayers. Aliquots of medium were collected from the receiver chamber after 0 min, 30 min, 60 min and 120 min and replaced with fresh HBSS; radioactivity in each sample was measured with a MicroBeta^2^ liquid scintillation spectrometer (Perkin Elmer, Milano, MI, Italy) and employed to calculate the apparent permeability coefficient (*P_app_*).

### 4.3. Bidirectional Transport Studies

The activity of ABC transporters was assessed by measuring the apical-to-basolateral (AB) and basolateral-to-apical (BA) fluxes of specific substrates. To this aim, cell monolayers were washed twice in HBSS and equilibrated for 30 min in the same solution (pH 7.4, 37 °C). Either the apical or the basolateral compartment (donor chamber) were then incubated in HBSS containing the labeled substrates Rhodamine123, ^3^H-estrone-3-sulphate and ^3^H-mitoxantrone for P-glycoprotein, MRP1 and BCRP, respectively, either in the absence or in the presence of the inhibitors PSC833, MK-571 and febuxostat for each transporter. Aliquots of the solution in the receiver chamber were collected after 0 min, 30 min, 60 min and 120 min and replaced with fresh HBSS. The fluxes were determined by measuring fluorescence with an EnSpire^®^ Multimode Plate Reader (Perkin Elmer), or radioactivity with MicroBeta^2^ liquid scintillation spectrometer. Data obtained were employed to calculate the apparent permeability coefficient (*P_app_*). The integrity of cell monolayers, verified by measuring TEER before and after the experiment, was not modified by the experimental procedures.

### 4.4. Calculation of P_app_

The apparent permeability coefficient (*P_app_*) of the tracer molecules was calculated according to the equation:

(1) $$P_{app}=\frac{\left(\frac{dQ}{dt}\right)}{\left(A\times C_{0}\times 60\right)}$$

where dQ/dt is the transport rate of the tracer, A is the filter surface area, C_0_ is the initial concentration of the tracer in the donor chamber, and 60 is the conversion from minutes to seconds.

Efflux ratio (ER) was calculated as the ratio between *P_app_* measured for BA and AB fluxes, while uptake ratio (UR) was calculated as the ratio between *P_app_* measured for AB and BA fluxes.

### 4.5. RT-qPCR Analysis

Total RNA was isolated with the GeneJET RNA Purification Kit and quantified through measurement of the A_260_/A_280_ ratio with a NanoDrop2000 Spectrophotometer (Thermo Fisher Scientific, Monza, MB, Italy). mRNA expression was then analyzed through RT-qPCR, as previously described [42]. Briefly, 1µg of total RNA was reverse transcribed with the RevertAid First Strand cDNA Synthesis Kit (Thermo Fisher Scientific) and qPCR was performed on 20 ng of cDNA by employing the StepOnePlus Real-Time PCR System (Thermo Fisher Scientific). The amount of *ABCB1*/MDR1 (NM_000927.4), *ABCC1*/MRP1 (NM_004996.4) and *ABCG2*/BCRP1 (NM_001257386.2), and the reference gene *RPL15* (ribosomal protein like 15; NM_001253379.2) were monitored by employing specific TaqMan^®^ Gene Expression Assays (Thermo Fisher Scientific; Cat# Hs00184500_m1, Cat# Hs01561512_m1, Cat# Hs01053790_m1, and Hs03855120_g1, respectively), according to the manufacturer’s instructions. The amount of the genes of interest was calculated relative to that of the reference gene using the formula:

(2) $$2^{\Delta Ct}\times 1000\;(\mathrm{where}\;\Delta Ct=Ct_{RPL15\;}-Ct_{gene\;of\;interest}).$$

### 4.6. Western Blot Analysis 

To monitor the expression of ABC transporter proteins, cell monolayers were washed with ice-cold phosphate-buffered saline (PBS) and lysed in Laemmli Sample Buffer (62.5 M Tris-HCl, pH 6.8, 2% SDS, 20% glycerol, 0.2 M dithiothreitol, DTT). Western blot analysis was then performed as previously described [43] by employing the following antibodies: anti-MDR1 (MDR1/ABCB1 (E1Y7B) rabbit mAb; Cat# 13342) and anti-MRP1 (MRP1/ABCC1 (D7O8N) rabbit mAb; Cat#14685) by Cell Signaling Technology (Euroclone, Pero, MI, Italy; 1:1000), anti-BCRP (ABCG2 (BXP-21); Cat# sc-58222) by Santa Cruz Biotechnology (DBA, Segrate, MI; Italy; 1:500) and α-tubulin (Anti-α-Tubulin, clone B-5-1-2; Cat# T5168) by Sigma-Aldrich (Merck, Milano, MI, Italy; 1:1000), employed as internal loading control. Horseradish peroxidase (HRP)-conjugated secondary antibodies (anti-rabbit and anti-mouse IgG) were provided by Cell Signaling Technology (1:10000). Immunoreactivity was visualized with Immobilon Western Chemiluminescent HRP Substrate (Merck, Milano, MI, Italy).

### 4.7. Immunocytochemistry and Immunohistochemistry

For immunocytochemistry, cell monolayers were rinsed in PBS, fixed with a 7-min incubation in methanol at −20 °C and permeabilized with a 20-min incubation in 0.15% Triton X-100 in PBS. After 1 h at 37 °C in blocking solution (5% of bovine serum albumin in PBS), cells were incubated overnight at 4 °C in blocking solution containing monoclonal antibodies targeting MDR1 or MRP1 (Cell Signaling Technology, 1:100), or BCRP (Santa Cruz Biotechnology, 1:100). At the end, monolayers were incubated for 45 min with secondary antibodies by Cell Signaling Technology (Alexa Fluor^®^ 488 conjugate; 1:400), then incubated with propidium iodide (Cell Signaling) for 10 min at 37 °C so as to stain nuclei. Lastly, permeable filters were detached from the culture inserts and mounted on glass slides with fluorescence mounting medium (FluorSave Reagent, Calbiochem, MerkMillipore, Milano, Italy). Slides were analyzed with a Zeiss^®^ 510 LSM Meta confocal microscope using a multi-track detection system and a 63× (Numerical Aperture, NA, 1.4) oil objective.

For immunohistochemistry (IHC), specimens obtained from bronchi or colon tissues of three healthy subjects were fixed in formalin immediately after sampling and embedded in paraffin. Histologic sections (4 µm) were incubated overnight at 4 °C in the presence of anti-MDR1 antibody (Cell Signaling Technologies, 1:100). After washing, sections were incubated for 60 min at 37 °C with anti-rabbit FITC conjugated secondary antibody (Jackson Laboratory). Nuclei were counterstained with 4′,6-diamidino-2-phenylindole (DAPI). Images were taken with an Olympus BX60 fluorescent microscope.

### 4.8. Statistical Analysis

The statistical analysis was performed using GraphPad Prism 7 (GraphPad Software, San Diego, CA, USA). All data were analyzed with a two tailed Student’s t-test for unpaired data, except for the measurement of TEER and mannitol/propranolol permeability and for expression of mRNA that were compared in the different cell models by means of one-way ANOVA with Bonferroni post test. *p* < 0.05 was considered significant.

### 4.9. Materials

Fetal bovine serum was purchased from EuroClone (Milano, Italy). ^14^C-mannitol (57.2 mCi/mmol), ^3^H-propranolol (18.6 Ci/mmol) and ^3^H-estrone-3-sulphate (49.19 Ci/mmol) were obtained from Perkin-Elmer while ^3^H-mitoxantrone (6.8 Ci/mmol) was obtained from Moravek Inc. All other chemical and reagents were from Sigma-Aldrich.

## Abbreviations

BCRP	Breast Cancer Resistance Protein;
HBSS	Hank’s Balanced Salt Solution;
MDR1	Multidrug Resistance 1;
MRP1	Multidrug Resistance-Associated Protein-1;
P_app_	apparent permeability coefficient;
TEER	Transepithelial Electrical Resistance.
